# ‘Enough is enough’: a mixed methods study on the key factors driving UK NHS nurses’ decision to strike

**DOI:** 10.1186/s12912-024-01793-4

**Published:** 2024-04-16

**Authors:** Daniel Sanfey

**Affiliations:** Centre for Medicine and Society, University of Freiburg, Fahnenbergplatz, 79085 Freiburg im Breisgau, Germany

**Keywords:** Nurses, Strikes, Industrial action, United Kingdom, National Health Service, Mixed methods

## Abstract

**Background:**

The UK National Health Service (NHS) is one of the largest employers in the world and employs around 360,000 registered nurses. Following a protracted pay dispute in December 2022 NHS nurses engaged in industrial action resulting in the largest nurse strikes in the 74-year history of the NHS. Initially it appeared these strikes were a direct consequence of pay disputes but evidence suggests that the situation was more complex. This study aimed to explore what the key factors were in driving UK NHS nurses’ decision to strike.

**Methods:**

A convergent parallel mixed methods design was used. The study was conducted throughout the UK and involved participants who were nurses working for the NHS who voted in favour of strike action. Data collection involved the use of an online survey completed by 468 nurses and 13 semi-structured interviews. Descriptive and inferential statistics were used for quantitative data analysis and a process of inductive thematic analysis for the qualitative data. The quantitative and qualitative data were analysed separately and then integrated to generate mixed methods inferences.

**Results:**

The quantitative findings showed that patient safety, followed by staff shortages, pay, and unmanageable work demands were the most important factors encouraging nurses’ decision to strike. The qualitative findings served to further the understanding of these factors particularly in relation to participants’ perception of the NHS and the consequences of inadequate pay and staff shortages. Three overarching and overlapping themes represented the qualitative findings: Save our NHS, Money talks, and It’s untenable. Integration of the findings showed a high level of concordance between the two data sets and suggest that the factors involved are interconnected and inextricably linked.

**Conclusions:**

The UK NHS is a challenging and demanding work environment in which the well-being of its patients is dependent on the well-being of those who care for them. Concerns relating to patient welfare, the nursing profession and the NHS played a large part in driving UK NHS nurses’ decision to strike. In order to address these concerns a focus on recruitment and retention of nurses in the NHS is needed.

**Supplementary Information:**

The online version contains supplementary material available at 10.1186/s12912-024-01793-4.

## Background

The United Kingdom National Health Service (NHS) is the seventh largest employer in the world [1] providing public health services for a population of around 67 million people [2]. Of the 1.4 million staff working for the NHS approximately a quarter of these are registered nurses [3]. Nurses are the backbone of the NHS providing hospital and community services and are often patients’ first and last point of contact when accessing care.

Nurses working for the NHS are paid according to a pre agreed pay and grading system decided upon by the UK Government with recommendations from an independent NHS pay review body. Research has shown that when taking inflation into account the average pay of NHS nurses has fallen in real terms by 8% between 2010/11 and 2021/22 [4], with the figure estimated at closer to 20% for more experienced nurses [5].

The Royal College of Nurses (RCN) is the largest nursing union in the world and represents around 405,000 registered nurses working in the UK [6]. Following a protracted pay dispute with the UK government, in October 2022 the RCN balloted its members working for the NHS on whether to take industrial action in the form of strikes. Despite the high threshold for success, with all ballots needing to be conducted by post and a 50% turnout and 40% vote in favour, the ballot was conclusive. NHS nurses voted in favour of strike action in the majority of NHS Trusts throughout the UK.^1^ In December 2022, for the first time in the RCN’s 106-year history their members engaged in strike action. The largest nursing strike in the 74-year history of the NHS.

On the surface it appears clear. NHS nurses were striking to secure better pay. This is supported by the most recent NHS staff survey [7] which found that only 25.6% of staff were satisfied with their level of pay. However, the staff survey also highlighted a number of other factors that indicate a high level of discontent, portraying the NHS as a stressful, demanding and unsatisfactory work environment. Furthermore, increasing numbers of nurses are leaving the profession due to health reasons, burnout and exhaustion [8], with additional nurses voicing their intent to leave because of high workload pressures and feeling undervalued [9]. This leads to the question: what are the key factors that have driven UK NHS nurses’ decision to strike?

Answering this research question is particularly pertinent at this time as the UK NHS is currently experiencing some of the greatest pressures in its history [10]. Waiting times are at an all-time high and record numbers of patients are waiting for treatment [11]. Not only are nurses engaging in strike action but also a plethora of other professions within the NHS including doctors, radiologists and physiotherapists; all of which only serves to exacerbate what is widely considered as an NHS in crisis [12]. At a time of widespread industrial action throughout the UK in which 2022 saw the highest number of working days lost to strikes for more than 30 years [13], determining the key factors driving UK NHS nurses’ decision to strike may serve to inform those concerned with prolonged and future industrial action, not just within the nursing profession and the NHS, but also the wider UK workforce.

### Literature review

A strike has been defined as ‘A temporary stoppage of work by a group of employees in order to express a grievance or enforce a demand’ (p.3) [14], Hyman [15] highlights that it is predominantly a calculated act and that the complete stoppage of work and its temporary and collective nature distinguish it from other forms of work-based protest.

Nursing strikes are a global phenomenon with incidences occurring in a diverse range of countries including America, Japan, Kenya, India, Australia and throughout Europe. In the UK nurses have a rich history of protest, but the incidences of strikes within the profession are few and far between. A limited number of empirical studies exist identifying factors that have driven nurses to go on strike. These include quantitative [16–18], qualitative [19–21], and mixed methods designs [22, 23]. Within these, issues relating to pay and working conditions predominate, but other factors such as intimidation from unions, failures of healthcare systems and addressing public perceptions of nurses were also found. What is notable is that none of these studies focus solely on factors driving nurses’ decision to strike, instead collecting data on a broad range of topics. This diverse approach may explain to some extent why they fail to facilitate a thorough understanding of the key factors driving nurses’ decision to strike. At present, it appears that there are no existing empirical studies focusing on nurse strikes within the UK, signifying a gap in the literature.

In addition to existing empirical studies there is a wide body of literature in the form of retrospective accounts that document and provide theoretical interpretations of individual and country specific nurse strikes [24–30]. By depicting the nurse strike within a historical, political, and professional context these accounts help to further illuminate the phenomenon and facilitate a much richer and deeper understanding. With this, we begin to appreciate the nurse strike as distinct from those within industrialised settings and as much a form of advocacy as that of self-preservation.

For any strike there are consequences. Whether they be for employers, workers, service users, the government, or for society at large. Within the healthcare environment there are concerns that a strike may have the additional consequence of compromising patient care. This has led some to denounce strikes by nurses citing them as immoral, unjustifiable [31] and wholly inappropriate [32]. Yet, it has been argued that such a stance fails to see the bigger picture and puts too much emphasis on the nurse/patient relationship [33].

Healthcare provision is a collective endeavour and whilst nurses have a professional responsibility to prioritise patient care and put the safety and wellbeing of those requiring care at the forefront of all they do [34, 35]; governments, employers and health policy makers also have a responsibility to facilitate an environment conducive to such an approach [36]. In situations where this does not happen it can be argued that to not stand up and take appropriate action would in itself be unethical [37] and antithetical to the standards required. It has therefore been posited that concerns around patient safety and standards of care can now be seen as one of the key driving factors for nurse strikes [26].

### Aims

The aim of this study is to explore what the key factors are driving UK NHS nurses’ decision to strike. The findings of this study can be used to inform government, employers, unions and health policy makers concerned with prolonged and future industrial action and stimulate a wider discussion around the demands of contemporary nursing and the challenges of working for the UK NHS.

## Methods

### Study design

**Fig. 1:**
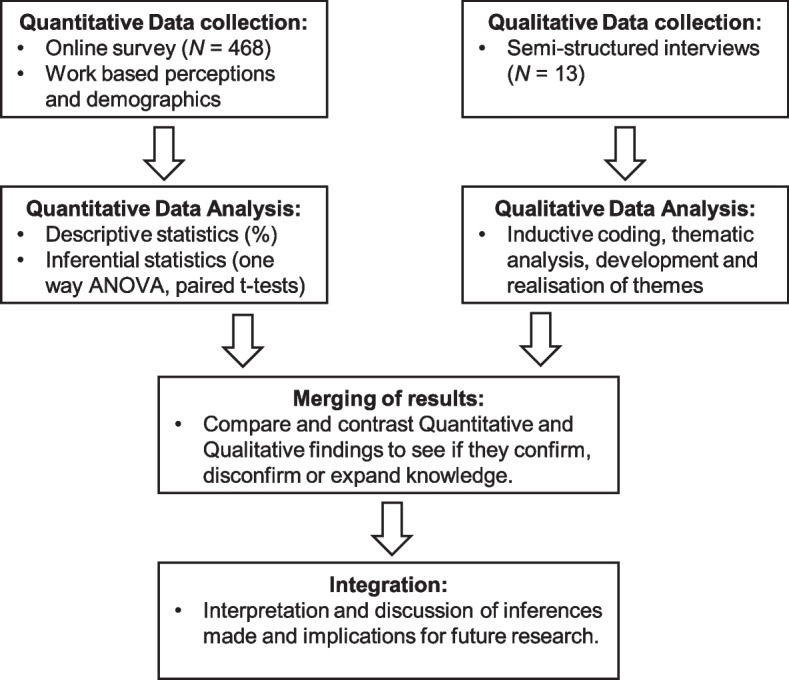
Convergent parallel mixed methods design used for study

A convergent parallel mixed methods design was used for the study to facilitate a detailed inquiry into the research question and enhance the validity of any inferences made. Quantitative and qualitative data were collected concurrently but separately, with equal importance given to each. The two data sets were then analysed independently, after which the results were merged and interpreted [38]. This approach helped to better understand the statistical trends associated with the nurse strikes whilst gaining a contextual understanding of the motivation and experiences that lay behind them. A summary of the study design can be seen in Fig. 1. The study is deemed exploratory in nature due to the lack of previous research on the topic within the UK and also to allow a certain amount of creativity and flexibility within the research methods used [39].

### Study setting and sampling

The study took place within the United Kingdom across all four nations of England, Wales, Scotland and Northern Ireland. The eligibility criteria included registered nurses working for the UK NHS who were also members of the RCN and voted in favour of strike action in the ballots conducted in October/November 2022 and/or May June 2023.

Participants were recruited for the quantitative methods through a combination of voluntary and convenience sampling. Nurses were notified of the study and invited to participate via the use of online nursing forums related to the RCN, social media sites (including Facebook, Twitter and LinkedIn), networking, and word of mouth. In addition, following approval from the local research and development (R&D) boards the study was advertised within two large NHS trusts in the south of England. This was achieved by distributing flyers and posters amongst the hospital wards. An email notification was also sent by the R&D department in one of the trusts. Further sampling was achieved on the picket lines outside two hospitals in Wales during the strikes in June 2023 with nurses informed of the study in person and provided with a QR code to access a survey.

Over 300,000 nurses were balloted in the first ballot on strike action in October/November 2022 [40]. The number of ballots completed and the proportion of nurses who voted in favour of strike action were not released and were not provided on request. However, with UK law [41] requiring a 50% response rate and a minimum of 40% voting ‘yes’ for strike action to happen we can assume that the actual population of nurses voting for strike action was at the very least 60,000^2^ nurses. A sample size of 384 participants was therefore deemed necessary in order for the sample to be representative. This was calculated using a basic prevalence sample size calculator with a confidence level of 95% and a margin of error at 5% [42].

Purposive and voluntary sampling was used for the qualitative methods. Potential participants were identified by scanning social media platforms for posts by nurses that implied they were in favour of and passionate about the strikes. Those nurses were then contacted, informed of the study and invited to participate (*n* 8). In addition, on hearing of the study a number of nurses came forward and volunteered their participation (*n* 5).

### Quantitative data collection and analysis

An online cross-sectional survey was designed and administered for the study (Additional file 1) and was made available via the digital survey platform Lamapoll [43]. Data was collected between the 21st April and 1st July 2023 and was analysed using IBM SPSS Statistics software v.29. Prior to data collection the survey was piloted and reviewed by 8 nurses who provided feedback. This resulted in minor adjustments in the wording for a single question and the subsequent removal of a sub-scale which was deemed unclear and lacking relevance.

The survey asked participants to select which factors they felt encouraged their decision to vote for strike action from a predetermined list. The list included seven factors which were compiled to reflect the most relevant points from the literature review, the stance of the RCN, and the current political climate within the UK. Descriptive statistics were used to depict how frequently each of the factors were chosen. In addition, participants were asked to rank those factors in order of importance. Means were calculated and compared. A one-way repeated measures ANOVA test was performed to determine whether the difference between the ranked levels of importance between factors was significant.

A number of the factors (staff shortages, pay and unmanageable work demands) were singled out for further exploration. This was done to try and gain an insight into the motivation lying behind each of those factors; that is, were those nurses more concerned with self-preservation and their own individual well-being (*self-motivation*), or were they more concerned with the well-being of the profession and the patients it cares for (*professional motivation*).

To determine the weighting towards the two constructs of self-motivation and professional motivation a series of Likert items were designed using a 5-point bipolar scale, ranging from *strongly disagree* to *strongly agree*. The items were divided into three subscales relating to each of the chosen factors. The three sub-scales combined had good reliability [44, 45] for both the Professional motivation construct (Cronbach’s α = .88; 6 items) and the Self-motivation construct (Cronbach’s α = .86; 6 items). Measures of internal consistency for the individual sub-scales can be found in Table 1. The order of questions within each sub-scale were varied to minimise acquiescent response bias [46]. The scales were numerically coded into interval data and grouped under their corresponding constructs. Paired t-tests were performed to determine whether the difference between the two constructs was statistically significant for each sub-scale.

**Table 1:** Internal consistency of sub-scales for individual constructs

Sub-Scale	Professional Motivation			Self-Motivation		
	*n*	Items	Cronbach’s alpha	*n*	Items	Cronbach’s alpha
Staff Shortages	440	2	.911	438	2	.926
Pay	409	2	.743	407	2	.805
Unmanageable Work Demands	349	2	.807	351	2	.829

Sub-scales labelled according to the corresponding factor that encouraged nurses’ decision to strike

Demographic data was obtained to inform what type of nurses participated in the survey and presented in tabular form using descriptive statistics.

### Qualitative data collection and analysis

Semi-structured interviews were conducted using an interview guide consisting of a range of questions and probes designed to elicit rich and insightful responses (Additional file 2). An additional set of probes were used for responses that complimented the factors listed within the survey so as to facilitate congruence between the data sets (Additional file 3). To allow the inclusion of participants from a broad geographical range interviews took place online via the video conferencing platform Zoom. They were conducted between the 23rd May and the 23rd June 2023. Interviews were conducted until it was felt that saturation of data was achieved; meaning, new data appeared to be repeating what was previously collected and thus, it was felt that further data collection was unlikely to add to the findings. The mean length of the interviews was exactly 50 minutes.

Thematic analysis of the data was conducted using the methods outlined by Braun and Clarke [47] with the help of MAXQDA 2022 data analysis software. These methods involved a 6-phase process. Phase 1 – *familiarisation,* began by a single researcher conducting the interviews and transcribing them verbatim. This helped to facilitate familiarity with and immersion of the data. An inductive approach was used for phase 2 - *generating initial codes,* in which coding of the transcripts was guided by the content of the data rather than any preconceived theoretical or epistemological perspectives. This phase generated over 90 interrelated and often overlapping codes which were sorted and organised using a mind map. Organising the codes in this way helped to see the relations between them and formed the beginnings of phase 3 – *searching for themes.* Initially this phase took on a rather positivist approach that saw the inception of themes based on the prevalence of codes and their semantic level context. However, a more interactive and organic approach developed in phase 4 – *reviewing themes,* where the initial set of themes were revised to ensure they really represented the coded extracts, as well as the story being told across the entire data set. It is here that the researcher’s subjective interpretation began to play a more influential role. Themes developed not just based on the data within the codes but on how they were perceived and understood by the researcher. This process gained momentum in phase 5 – *defining and naming themes* where the essence of each theme, how they related to one another and the story that they told was fully realised. Phase 6 – *producing the report* saw the outcome of this process in which the qualitative results tell a story that reflects the coming together of the experiences, meaning and reality of participants with that of the understanding, values and skills of the researcher.

### Mixed methods analysis

Quantitative and qualitative data were integrated at the interpretation and reporting level. The key findings of the quantitative data were presented alongside qualitative data using a joint display table. This approach helped to merge the data in a more direct way and facilitate a better understanding of the mixed methods meta-inferences [48].

### Ethical considerations

Ethical approval was granted by the University of Freiburg’s ethical research committee (Application no. 23–1126-S2). All surveys were completed anonymously and informed consent gained from all participants. Participants who partook in the interviews were provided with a participant information sheet and asked to sign a consent form prior to being interviewed. The interviews were anonymised during transcription with all identifiable data subsequently deleted. All data was held and stored in accordance with the UK Data Protection Act of 2018. Participation was completely voluntary, and no financial incentives made.

## Results

Five hundred forty-four nurses responded to the survey. Those that did not fulfil the eligibility criteria or provided an insufficient amount of data were discarded, resulting in 468 completed surveys included in the analysis. Thirteen participants were recruited for the semi-structured interviews. The demographics and work-based characteristics for the quantitative and qualitative samples are displayed in Tables 2 and Table 3 respectively. Female nurses working in hospital settings with adult patients predominated. There was a broad range of experience across the two data sets with the majority of nurses having trained in the UK. Demographics for RCN membership were not available to draw comparisons with; however, the sample is broadly proportional to that of the UK nursing register with regards to age, gender and type of nursing. It is underrepresented by mental health nurses and those who trained outside of the UK [49].

**Table 2:** Demographics of participants of quantitative methods

Characteristic	n	*%*
Age		
18–24	10	2.1
25–34	90	19.2
35–44	111	23.7
45–54	144	30.8
55–64	93	19.9
65 and over	8	1.7
Gender		
Female	397	84.8
Male	56	12.0
Non-Binary	1	0.2
Prefer not to say	3	0.6
Main work setting		
Hospital	356	78.1
Community	82	17.5
Both	18	3.8
Type of nursing		
Adult	351	75.0
Paediatric	70	15.0
Learning Disability	5	1.1
Mental Health	31	6.6
Current band working at ^a^		
5	131	28.0
6	148	31.6
7	134	28.6
8	44	9.4
Location of nurse training		
Inside the UK	428	91.5
Outside the UK but within Europe	12	2.6
Outside of Europe	17	3.6
Years of experience as registered nurse		
0–5	75	16.0
6–10	65	13.9
11–15	48	10.3
16–20	62	13.2
21 and over	207	44.2

*N* = 468. The totals do not equate to this due to missing values

^a^ Band according to Agenda for Change pay scales

**Table 3:** Demographics of participants of qualitative methods

Characteristic	n	*%*
Gender		
Female	10	76.9
Male	3	23.1
Main work setting		
Hospital	8	61.5
Community	4	30.8
Both	1	7.7
Type of nursing		
Adult	10	76.9
Paediatric	2	15.4
Mental Health	1	7.7
Current band working at ^a^		
5	3	23.1
6	5	38.4
7	3	23.1
8	2	15.4
Geographical location		
North West England	2	15.4
South East England	4	30.8
London	3	23.1
South West England	2	15.4
West Midlands England	1	7.7
Scotland	1	7.7

*N* = 13

^a^ Band according to Agenda for Change pay scales

### Quantitative results

**Fig. 2:**
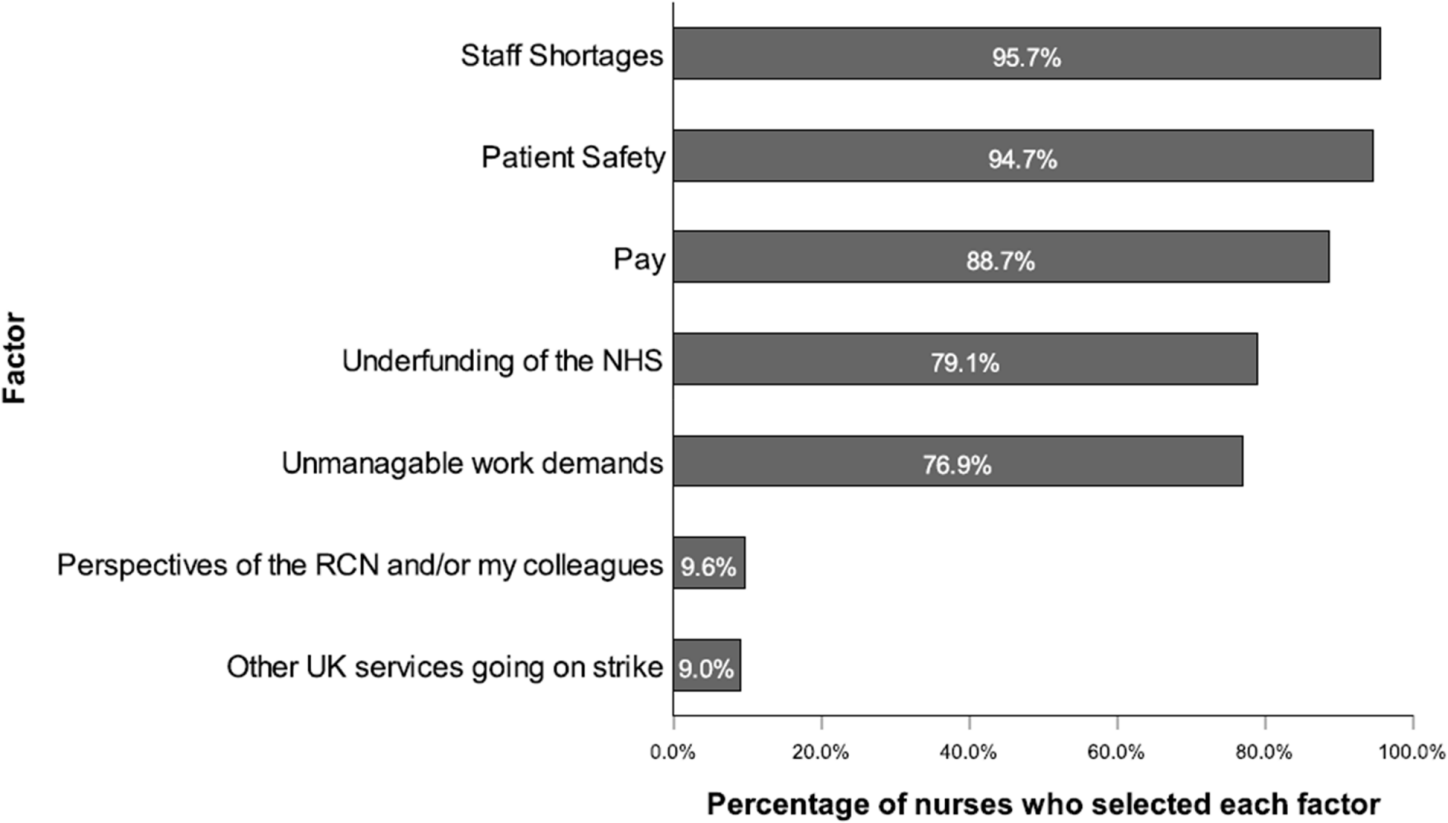
Factors that encouraged participants’ decision to strike. *Note*. *N* = 468

The factors that encouraged nurses’ decision to strike are displayed in Fig. 2. The mode number of factors chosen was 5, in which Staff shortages and Patient safety were the most frequently cited.

Nurses indicated that patient safety, followed by staff shortages were the most important factors that encouraged their decision to strike. The ranked means and standard deviations for the level of importance ascribed to each factor are presented in Table 4. A one-way repeated measures ANOVA found that the difference between the level of importance for the factors was significant at the .05 alpha level. Wilks’ Lambda = .04, *F* (6, 462) = 2149.69, *p* < .001, multivariate partial eta squared = .97. However, post-hoc pairwise comparisons with a Bonferroni adjustment indicate that the difference was not significant between each level of ranking. Those that were significant are highlighted in Table 4.

**Table 4:** Ranked means showing level of importance of factors encouraging participants’ decision to strike

Factor	*M*	*SD*
Patient Safety	5.85	1.67
Staff Shortages	5.57_a_	1.49
Pay	4.32_a_	2.01
Unmanageable work demands	3.87_b_	2.31
Underfunding of the NHS	3.20_b,c_	1.94
Perspectives of the RCN and/or my colleagues	.35_c_	1.09
Other UK services going on strike	.21	.71

*N* = 468. Levels of importance were ranked from 1 (*least important*) to 7 (*most important*), with those factors not chosen coded as 0 (*not important*). Only 1 factor could be chosen per rank. Factors are displayed in descending order with higher means indicating higher level of importance. Means sharing subscripts are significantly different from each other at *α* = .05. The *p* value for each was *p* = <.001

**Fig. 3:**
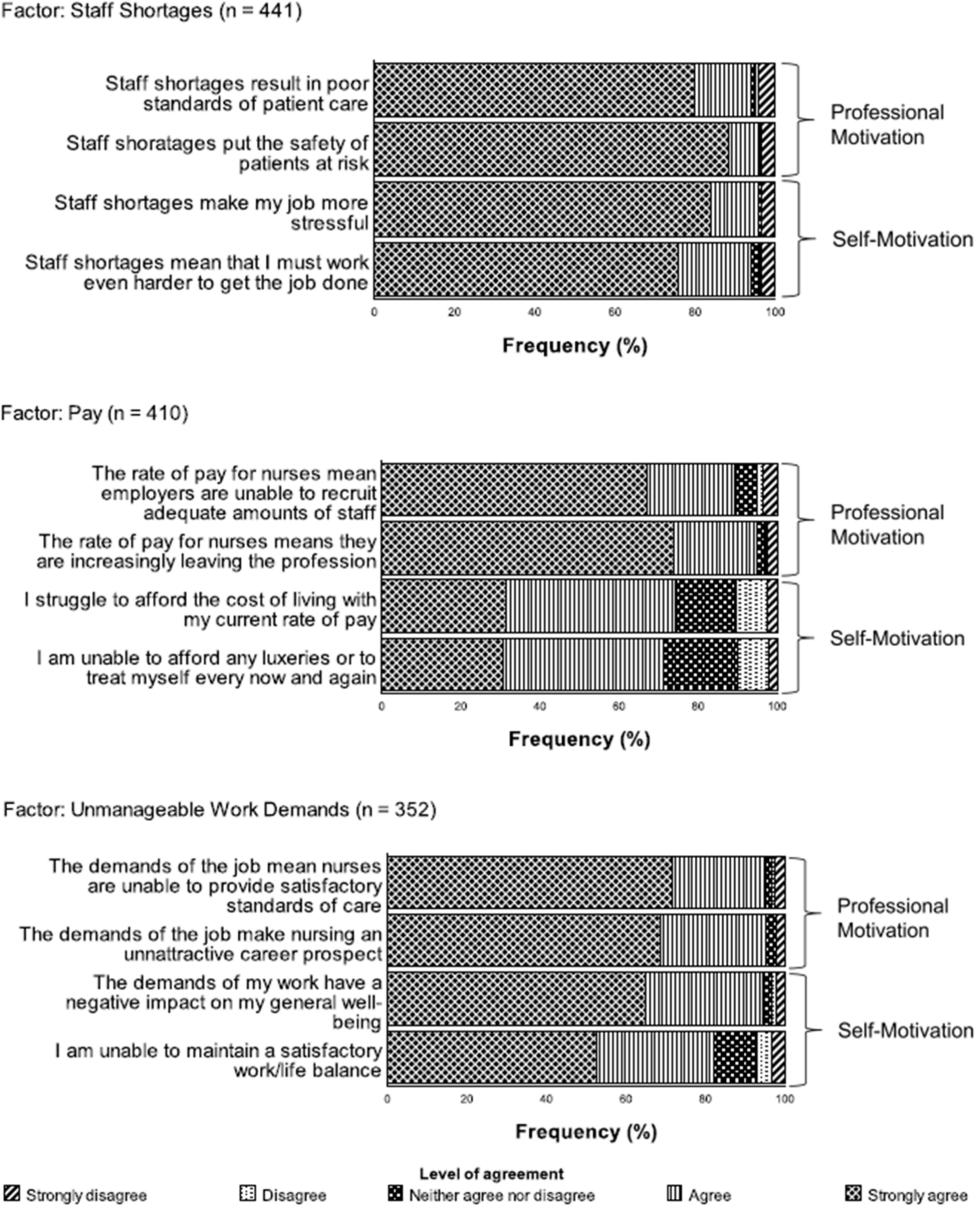
Likert sub-scales showing individual items and their relation to the constructs professional motivation and self-motivation. *Note*. Order of items presented to facilitate easy understanding of how the items relate to the constructs of Professional Motivation and Self-motivation. The order of items within the survey was different

Responses to the Likert sub-scales and the level of agreement that nurses had to the individual items are presented in Fig. 3. The results of the paired t-tests (Table 5) indicate that those who cited pay and unmanageable work demands as factors that encouraged their decision to strike were significantly more professionally motivated than self-motivated (*α* = .05). However, it should be noted that the effect size, whilst moderate for pay was small for unmanageable work demands. The difference between the level of professional motivation and self-motivation for those who cited staff shortages as a factor that encouraged their decision to strike was not statistically significant.

**Table 5:** Differences between the constructs professional motivation and self-motivation according to their corresponding factor

Factor	Professional Motivation		Self-Motivation					
	*M*	*SD*	*M*	*SD*	*df*	*t*	*p*	*Cohen’s d*
Staff Shortages	9.44	1.58	9.36	1.58	436	2.01	.111	0.10
Pay	9.11	1.54	7.83	1.83	405	14.91	<.001	0.74
Unmanageable Work Demands	9.20	1.42	8.79	1.68	346	6.19	<.001	0.33

*p* values adjusted using Bonferroni adjustment method

### Qualitative results

The process of thematic analysis identified three overarching and overlapping themes which were selected to represent the data. These themes included: Save our NHS, Money talks, and It’s untenable.

#### Save our NHS

The state of the NHS was reflected upon throughout the interviews. Participants were passionate about the NHS and its ability to provide high standards of safe and effective care, free at the point of need. However, there was a recognition that the NHS was failing as an institution, the injustice of which was palpable and articulated by the following comment:

I don't understand, genuinely don't understand why people aren't rioting because of the state of the NHS. We are going to lose this incredible thing… It's just, I mean, I feel quite emotional. It’s just shocking. It's just shocking that it's happening.

Participant 2

Witnessing the decline of the NHS seemed to elicit a sense of loss and foreboding in participants. One nurse explained how this had evolved into a sense of shame at what it has become.

I was very proud going back years ago to put on a uniform, to enter that building and start my shift. And I was proud to tell people that I worked for the NHS. And I'm not anymore. I'm embarrassed by it. I'm embarrassed by the care that we give. I’m embarrassed by the treatment that some of the patients get. It's heart breaking, it really is.

Participant 10.

And yet, these feelings seemed to stem, not from an idealistic view of what the NHS should be or how it should be run, but rather from the lived experience of providing frontline care on a day-to-day basis. This results in a visceral understanding that nurses are not just the providers of care but also the recipients of care, along with their families, loved ones, and the nation as a whole. For example, on reflecting on caring for a dying patient participant 6 acknowledged “That could be me one day.” In addition, whilst talking about the poor standards of care she had witnessed, participant 13 expressed “I’m worried about the care my parents are going to receive, I’m worried about the care I’m going to receive in the future!” Participant 5 spoke about living with a congenital heart condition, needing regular specialist review, extensive surgery and a costly hospital stay. He concluded “I’ve really benefited from the NHS, as an end user and also as an employee. I think it’s a great institution and I think it needs to continue.”

Nurses’ decision to strike could therefore be seen as a call to arms in response to the witnessed decline of the NHS and all that it entailed. The value and appreciation that nurses hold for the NHS comes with a real sense that it is worth fighting for. The decision to strike was seen to play an important part in that fight. Participant 12 highlighted this point in saying:

It really is about the health of all people in the UK and the future of what that's going to look like. And it's not looking good, you know, from where we're at just now. If we don't fight, I believe there's a real possibility we could lose the NHS.

#### Money talks

It was widely felt throughout the interviews that the rate of pay that nurses receive does not reflect their level of expertise, professional development, and the responsibility that comes with the job. The following nurse discussed this in relation to her own professional development.

If I did my nurse prescribing in a couple of years, which is a possibility, it's not going to get me any more pay. And the level of responsibility that comes with that… We're a very responsible profession, you know, breaking bad news, seeing things that the ordinary general public wouldn't even dream to see… And yet, we're not recognised financially, or with the respect as a profession that we deserve.

Participant 11

Participants throughout the interviews felt that the nursing profession was undervalued and underappreciated, especially by the government and thus, their decision to strike was an attempt to highlight this. This sentiment was particularly pertinent in relation to the recent COVID pandemic. Participants reflected upon the sacrifices they made during the pandemic and the discrepancy of being hailed as heroes by the government one day, to receiving yet another below inflation pay rise the next. This served to exacerbate the feeling of being undervalued as highlighted in the following excerpts:

My husband had a heart attack during the pandemic, I couldn't visit him. But I was still going to work. I was in this building. But I couldn't go and see him. There are huge, huge sacrifices made by all of us. Four of my colleagues died, and we're not even worth a real time pay increase!

Participant 2.

Yes, let's all stand out on our doorsteps and clap and bang our pans for the wonderful people who are doing a wonderful job. But actually, when you want a decent wage, we're not going to give that to you.

Participant 5

The level of pay that nurses receive was therefore perceived as a measure of the value and appreciation ascribed to the profession. In addition, better pay was viewed as a vital tool in incentivising people to become nurses and to work in the NHS. This point was made by Participant 4 who explained:

I don't personally care about the pay. For me at least as an individual… But I do care about pay for my colleagues and the wider NHS, is it 47,000 nursing vacancies? They're not going to get filled with shoddy pay. There needs to be an incentive to be a nurse at the moment.

The pursuit of better pay was a key factor driving nurses’ decision to strike because better pay was seen as integral to addressing the ongoing recruitment and retention crisis of nurses within the NHS.

#### It’s untenable

The recruitment and retention crisis, and it’s resulting staff shortages was frequently cited by participants as being the root of the problem and fundamental to their decision to strike. Staff shortages result in nurses having to take on an additional workload to meet the needs of patients. As participant 7 explains, “It’s not doable. You’re having to work twice as hard… You’re having to do several people’s jobs.” Participants spoke of how staying late after work and working through their breaks to try and keep on top of the workload was an everyday occurrence. The relentless pressure and responsibility of the job is at times overwhelming and the impact on individual nurses seen as untenable. As participant 13 pointed out.

We're not designed to be in flight mode all the time, are we? And if we don't get respite, then we're in trouble and that's what we're seeing on our work force right now in terms of how people feel, burnout, wanting to leave, going off sick….

This was also reflected upon by participant 10 who spoke about her own experiences of being burnt out from work and how this impacted her.

I ended up being off for three months… I was at the point where I didn’t want to be a nurse anymore, I didn’t want to be in my marriage. I wanted to walk out of my home, my children, my…. I just wanted to pick-up and walk out of my life.

With this we see that the morale of nurses working in an environment that is chronically understaffed is persistently under threat. As participant 1 lamented, “it makes you feel inadequate. It makes you feel that you’re not doing your job as well as you should be.” A point further elaborated on by participant 11 who noted: “nurses can’t be the nurses that they want to be. You know, they’re feeling disappointed with themselves, they’re feeling let down, they feel that they have failed.” All of this results in more and more nurses leaving the NHS or the profession completely, which only serves to exacerbate the problem of staff shortages. The decision to strike was effectively a way of nurses saying, “enough is enough, this cannot go on!”

Despite the strains of the job, it is interesting to note however that participants largely considered the real consequences of staff shortages to be suffered by patients. With increasing workloads and high patient to nurse ratios nurses’ ability to provide even the most basic standards of care are compromised. They are often faced with difficult decisions on prioritising and allocating care; things get missed, mistakes happen, and treatments and care are not provided in a timely fashion. This compromises the safety of patients and results in them coming to harm. A point stressed by participant 13.

I have seen, and I have experienced patients having poor health outcomes, or poor experiences as a result of not being able to deliver the care that we know we can deliver. And that's because of circumstances such as short staffing, and people being off long-term sick with stress.

It seems then that nurses’ decision to strike was a cry for help, not just for nurses working within the NHS, but for the very patients it aims to serve.

### Integration of quantitative and qualitative results

Integration of the quantitative and qualitative findings show a high level of concordance between the two data sets. Table 6 provides examples of how the qualitative findings not only confirmed the key quantitative findings (*confirmation*) but also served to expand the understanding of them (*expansion*). No incidences were found where the two sets of findings contradicted each other (*disconfirm*).

**Table 6:** Integration of quantitative and qualitative findings to facilitate mixed methods meta-inferences

Key Quantitative Findings	Related Qualitative Theme and Supporting Quotes	Mixed Methods Meta-Inferences
Data indicates that patient safety was the most important factor encouraging nurses’ decision to strike (*M *= 5.85, *SD *= 1.67), followed by staff shortages (*M *= 5.57, *SD *= 1.49). The level of importance between the two factors was not found to be significant (*p *= .072).	*It's untenable:* “It is mostly about patient safety, and I do really feel that patients’ safety is a massive issue at the moment…” (Participant 1). “There's so many things to patient safety. And having more staff makes it safer.” (Participant 6). “My main reasons are safety of patients, I want safer staffing levels”. (Participant 10).	*Expansion:* Staff shortages pre-cede patient safety and the two factors are inextricably linked. Staff shortages result in not enough nurses to adequately care for patients and thus, patient safety becomes compromised. In addition, staff shortages put undue pressure on nurses making the job untenable. This results in nurses increasingly leaving the profession and the NHS which further exacerbates staff shortages and compromised patient safety.
Those participants that cited pay as a factor that encouraged their decision to strike were significantly more professionally motivated than self-motivated (*p *= <.001).	*Money talks:* “Yeah, so for me I do okay, my salaries alright. It's not about me. It’s about the system as a whole.” (Participant 5) “Yeah, I'm talking about the profession as a whole. I mean personally I'm fine.” (Participant 9). “No, it's actually not about my personal struggles financially. For me it's a much bigger issue than that.” (Participant 12). “I'm not doing this for me… it’s for the new nurses coming through. My heart breaks for them coming through on their big debts… I don't think they should have to pay for their training.” (Participant 3). “So, first of all the students lost their bursaries, and then they had to pay student loans… So on that basis I decided to strike. It wasn't for me, it was for encouraging young people”. (Participant 2).	*Confirmation:* Pay was a factor that encouraged participants decision to strike primarily because an increased level of pay would serve as an incentive to attract people to join the profession and to stay in the profession, rather than for individual monetary gain. Increasing the rate of pay for nurses was therefore seen as the first step toward, and integral to addressing the recruitment and retention crisis. *Expansion:* The qualitative findings helped to identify a further insight into pay and professional motivation that wasn’t captured by the quantitative findings. This was in relation to student and newly qualified nurses. Increased pay was deemed especially important for these groups due to the costs of tuition fees and the vast debts that student nurses incur to do their training. Participants stressed that student nurses shouldn’t have to pay for tuition fees and the abolition of the student nurse bursaries in 2017 was widely criticised.
Those participants that cited unmanageable work demands as a factor that encouraged their decision to strike were significantly more professionally motivated than self-motivated (*p *= <.001).	*It's untenable:* “The staff that we do have are being utilised so much that they're burning out… it's no wonder they're leaving.” (Participant 10). “I'm talking about retention, absolutely. So many nurses who potentially might have worked a little bit longer towards retirement… leaving early or, going to work elsewhere in a job that's less stressful, less responsibility. You know, so we are haemorrhaging those experienced nurses.” (Participant 11).	*Confirmation:* Unmanageable work demands encouraged participants decision to strike because it was viewed as an important reason why nurses are leaving the profession and the NHS. Making the demands of the job more manageable was therefore seen as integral to addressing the retention crisis and making nursing a more attractive career prospect.

## Discussion

This mixed methods study offers valuable insights into the key factors driving UK NHS nurses’ decision to strike. The quantitative findings identify that patient safety, followed by staff shortages and pay were the most important factors. The qualitative findings support these findings and further enhance our understanding of them. Mixed methods inferences suggest that the factors driving UK NHS nurses’ decision to strike are complex, interconnected and inextricably linked.

What is notable from the findings was that two factors: perspectives of the RCN and/or my colleagues and other UK services going on strike were deemed the least important factors and cited by less than 10% of participants. In addition, they did not arise within the qualitative data. This suggests that the decision to strike by participants was made with a high level of autonomy and was largely independent of the widespread industrial action taking place within the UK during that time.

In contrast to other empirical studies conducted on nurse strikes outside of the UK [16–18, 21–23] this study found that pay was not the most cited factor encouraging nurses’ decision to strike. Due to these studies varying considerably in their aims, context and methodological profiles it is difficult to draw any definitive conclusions as to why this difference occurs; however, it suggests that factors driving nurses’ decision to strike are context specific and reflective of differing cultural and economic environments.

Although pay was not found to be the most important factor, the qualitative findings indicate that it still plays an integral role in encouraging nurses’ decision to strike. In part, this is because it was seen as an indicator of how valued and appreciated the nursing profession is. West et al. [50] argue that this sense of value is essential for nurses’ well-being and their ability to deliver high-quality care. The finding that nurses perceive pay as a measure of value is supported by Clayton-Hathway et al. [51] who go on to suggest that the lack of value ascribed to the nursing profession, and its resulting low pay is rooted in the perception of nursing as ‘women’s work’ and indicative of the patriarchal society historically found within the UK. This concept of gender disparities in relation to pay is compelling and challenges the assumption that low pay is simply to do with a lack of funds. It suggests that further research on the qualitative determinants of nurses pay would be valuable.

Within this study it was found that there was a high level of both self and professional motivation behind the factors driving participants’ decision to strike, but it was the latter that predominated. The concept of professional motivation being a driving force in nurses’ decision to strike is supported by accounts of nurse strikes both within the UK [25, 52] and outside [19, 24, 26, 28]. Briskin [24] referred to it as ‘the politicisation of caring’, a theory closely aligned to Hart’s [25] ‘clinical militancy’. However, there is a danger in adopting such terminology that we are merely conforming to the stereotypes around industrial action and failing to adequately reflect the nuances of the nurse strike. The findings of this study indicate a softer, more considered approach by nurses that is deeply rooted in a sense of moral justice and duty of care. With this understanding one is compelled to rethink the depiction of the strike as a form of self-gratifying militancy, to that of a legitimate act of compassionate care [53].

The finding that professional motivation plays a significant role behind the factors driving UK NHS nurses’ decision to strike is important as it can be used to garner public support for future nurse strikes and better inform those in opposition to them. In addition, it can be used by the RCN to reflect upon their communication strategies and ensure they adequately reflect the perceptions of their membership; furthermore, it may serve to challenge those accounts by media outlets that portray the strikes to be driven solely by individual monetary gain. A suggestion for further research could therefore be to conduct a content analysis on the media coverage of the strikes and compare the findings with that of this study. This could provide valuable insights into the validity of the mainstream media’s interpretation of strikes and the role it plays in influencing public opinion.

**Fig. 4:**
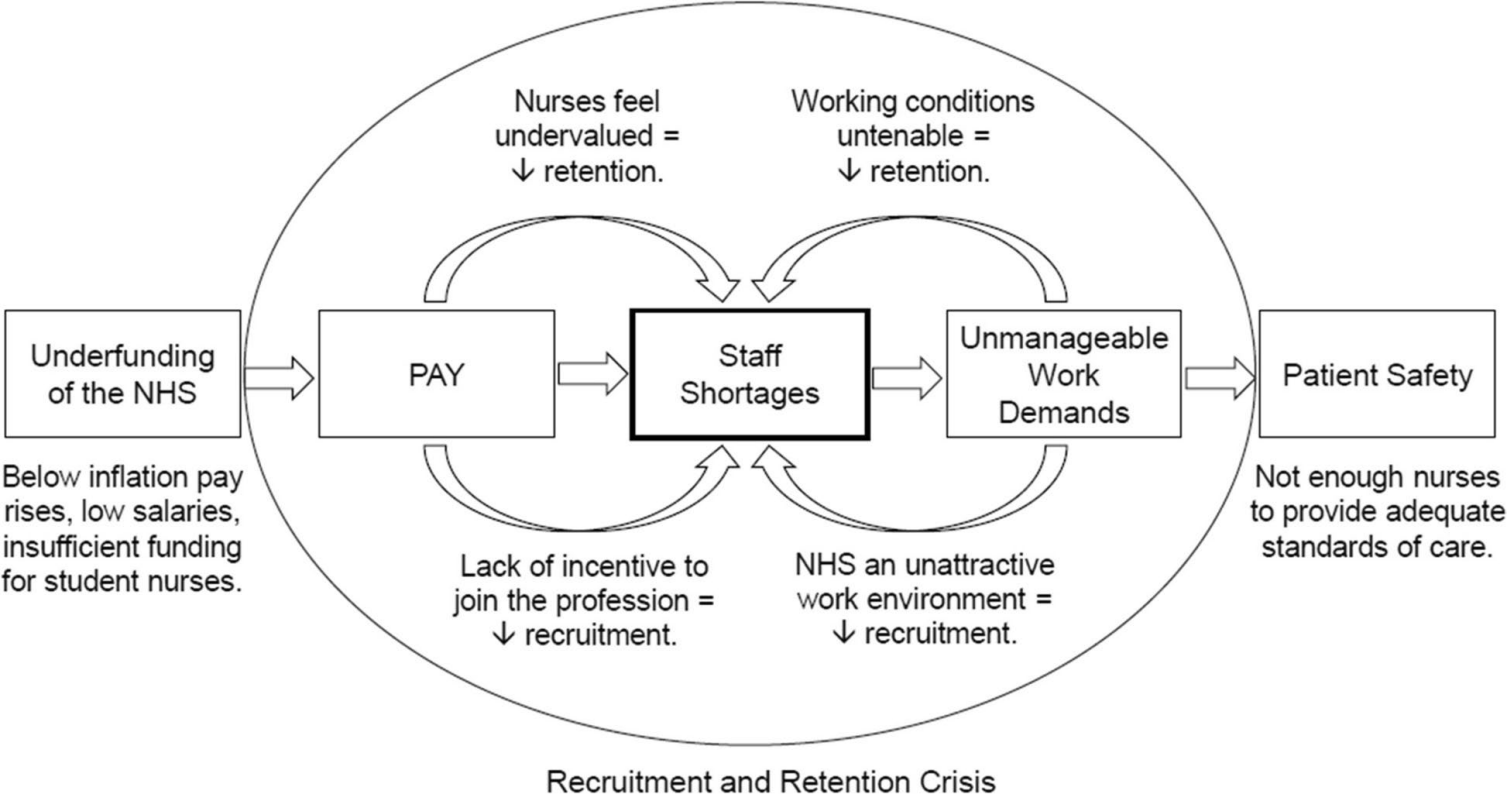
Interconnectedness of factors drawn from mixed methods inferences. *Note*. This model shows how factors encouraging nurses’ decision to strike lead into one another and are centred around staff shortages. The interplay of pay, staff shortages and unmanageable work demands creates a vicious cycle that manifests as a recruitment and retention crisis, resulting in compromised patient safety

The mixed methods inferences of this study help us to understand that the factors driving UK NHS nurses’ decision to strike are complex, multifaceted and inextricably linked. Figure 4 provides a conceptual model of these inferences and summarises the interconnected nature of the factors.

### Limitations

The findings of this study should be judged within the context of its limitations. First of all, it should be noted that this study was conducted by a single lone researcher who is also a registered nurse working for the UK NHS and a member of the RCN. Whilst every attempt was made to reduce bias and provide a true representation of participants perspectives the lack of investigator triangulation leaves the study susceptible to observer bias. In particular, the validity of the qualitative findings would have been enhanced by a second reviewer confirming the selection of and allocation of codes, and the generation of themes.

A further limitation can be found in the sampling methods used. The use of voluntary sampling means that the findings are likely to be subject to self-selection bias and thus less representative of those nurses who were less forthright about their decision to strike. Furthermore, a large proportion of participants were recruited via social media meaning that the study may not adequately reflect the views of those nurses who do not use social media. Data collection began approximately 5 months after the initial ballot in which nurses first voted to strike. It may have been that by this time there was an element of strike fatigue resulting in an unwillingness to participate and engage with the study. Had the data collection happened sooner it may have helped to minimise response bias and encourage greater participation.

This notion of strike fatigue may also explain to some extent why the RCN failed to secure a further strike mandate following the completion of this study. In focusing on the key factors that drove nurses’ decision to strike this study fails to adequately portray how those decisions, and the volition to strike may change over time.

Although the results of this study are compelling it is important to recognise that an element of social desirability bias may have played a part. Participants may have felt drawn toward emphasising those factors that portrayed them as striking for the greater good so as to uphold the reputation of the profession and justify the act. Although it is not possible to quantify to what extent social desirability bias played a role it should be taken into consideration when interpreting the results.

In keeping with the exploratory nature of this study a novel approach was used in the survey design. Due to the lack of previous research in this area and the absence of a strong theoretical foundation in relation to the constructs used, there is a danger that the survey lacks construct validity. The survey would therefore benefit from greater scrutiny in the form of expert opinion review, further research and refinement with the use of factor analysis.

## Conclusions

This mixed methods study has facilitated an exploration into the key factors driving UK NHS nurses’ decision to strike leading up to and during the industrial action of 2022/23. The findings identify that factors relating to patient safety, staff shortages, pay and unmanageable work demands were key, and that there was a strong sense of professional motivation lying behind them; that is, participants concerns around the welfare of patients, the nursing profession and the NHS often came before that of their own.

In adopting a mixed methods design this study helps to highlight that the factors driving UK NHS nurses’ decision to strike do not stand in isolation and therefore, a holistic and multifactorial approach to addressing them is required. Nurses’ concerns around recruitment and retention and the implications of staff shortages need to be taken into consideration. Perhaps more importantly however, this study demonstrates that the NHS is a challenging and demanding work environment, and that the well-being of its patients is dependent on the well-being of those who care for them. If nobody cares for the carers the process of healthcare delivery breaks down. Thus, one can consider these nurse strikes as a movement, a movement toward *putting the care back into care*.

This study paves the way for future research on nurse strikes and could also be used to inform research into other healthcare related professions engaged in industrial action. Further research looking at the factors driving nurses’ decision to strike is required to confirm the validity of these findings and also to develop the constructs of self and professional motivation in relation to strikes. In addition, research looking into the perspectives of the mainstream media on nurse strikes and the determinants of nurses’ pay would offer valuable insights and increase our understanding of the nurse strike.

### Supplementary Information

**Supplementary Material 1.**

**Supplementary Material 2.**

**Supplementary Material 3.**

## Data Availability

The datasets used and/or analysed during the current study are available from the corresponding author on reasonable request.

## Abbreviations

R&D: Research and Development
RCN: Royal College of Nursing
NHS: National Health Service
UK: United Kingdom

## Footnotes

1 NHS Trusts are organisations that provide healthcare services on behalf of the NHS within a given specialisation or geographical area. The majority of nurses working for the UK NHS are employed directly by an NHS Trust.

2 The actual figure is likely to be far higher but a further increase in population size does not influence the sample size calculation.
