# Supplementary material for: ‘Enough is enough’: a mixed methods study on the key factors driving UK NHS nurses’ decision to strike
Source: BMC Nurs. 2024 Apr 16;23:247. doi: 10.1186/s12912-024-01793-4 (PMC11020814; doi:10.1186/s12912-024-01793-4)
Supplement: Supplementary file 1 — Supplementary Material 1. [file 12912_2024_1793_MOESM1_ESM.docx]

**Additional File 1**

Format: Microsoft Word Document (.docx)

Title: Cross-Sectional Survey

Description: Survey used for collecting quantitative data on the perspectives of nurses who voted in favour of strike action.

**
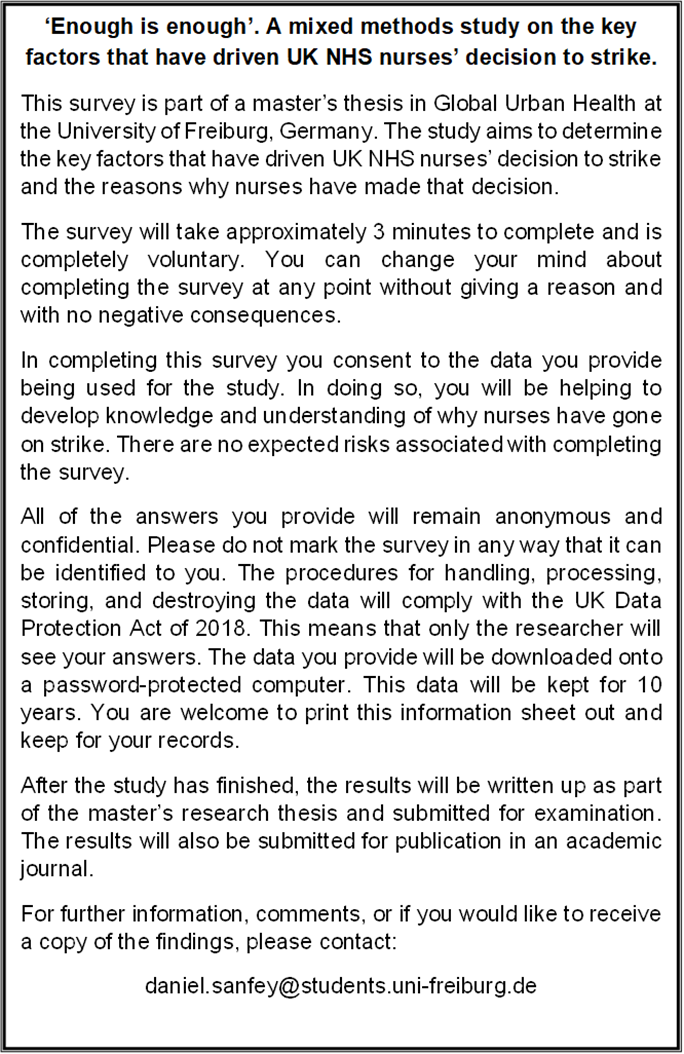
**
