# Supplementary material for: ‘Enough is enough’: a mixed methods study on the key factors driving UK NHS nurses’ decision to strike
Source: BMC Nurs. 2024 Apr 16;23:247. doi: 10.1186/s12912-024-01793-4 (PMC11020814; doi:10.1186/s12912-024-01793-4)
Supplement: Supplementary file 2 — Supplementary Material 2. [file 12912_2024_1793_MOESM2_ESM.docx]

**Additional File 2**

Format: Microsoft Word Document (.docx)

Title: Semi Structured Interview Questions and Potential Probes

Description: Interview guide for semi-structured interviews collecting qualitative data on the perspectives of nurses who voted in favour of strike action.

| **Question** | **Potential Probes** |
| --- | --- |
| 1. Can you tell me a bit about your background working as a nurse? How long, where, what type of nursing etc? | *Do you feel the job has changed over that time?* |
| 2. I would like you to think back to when you were asked to vote on whether to strike or not. Can you tell me how you felt making that decision? | *Can you tell me a bit more about that?*  *Was it an easy decision, a hard decision?* |
| 3.What were the main reasons that drove your decision to strike? | *Can you give me some examples of how that impacts you (personally/professionally)?* |
| 4. What changes do you think need to be made to improve the situation? | *Can you be more specific?*  *Ask for concrete examples.* |
| 5. Have your feelings changed with regards to going on strike since the first ballot? | *If so, how.* |
| 6. What advice would you give someone who was unsure whether to strike? |  |
| 7. Where do you see the nursing profession in ten years’ time? | *Where do you see your career in ten years’ time?* |
