# Supplementary material for: ‘Enough is enough’: a mixed methods study on the key factors driving UK NHS nurses’ decision to strike
Source: BMC Nurs. 2024 Apr 16;23:247. doi: 10.1186/s12912-024-01793-4 (PMC11020814; doi:10.1186/s12912-024-01793-4)
Supplement: Supplementary file 3 — Supplementary Material 3. [file 12912_2024_1793_MOESM3_ESM.docx]

**Additional File 3**

Format: Microsoft Word Document (.docx)

Title: Additional Probes for Semi-Structured Interviews Relating to Factors Driving Nurses’ Decision to Strike

Description: Additional probes used in semi-structured interviews to facilitate congruence between quantitative and qualitative data sets.

| **Factor** | **Potential Probes** |
| --- | --- |
| Patient safety | *What do you mean by patient safety?*  *Can you give examples of times when you have felt it to be unsafe?* |
| Staff shortages | *How does being short staffed impact you and your work?* |
| Pay | *Is it to do with the amount of pay you receive personally or the profession as a whole?* |
| Unmanageable work demands | *How does it make you feel?*  *How does it impact your work?*  *Can you give examples?* |
